# Patient-Reported Quality of Life and Fatigue After a Theory-of-Planned-Behavior-Informed Multicomponent Exercise Intervention During Systemic Treatment for Colorectal Cancer: A Secondary Analysis of a Randomized Trial

**DOI:** 10.3390/healthcare14213771

**Published:** 2026-11-04

**Authors:** Zhenyu Liao, Xiaomin Chen, Qian Wu

**Affiliations:** 1 Department of Nursing, Shanghai Tenth People’s Hospital, Tongji University School of Medicine, Shanghai 200072, China; 2632553@tongji.edu.cn; 2 Nantong First People’s Hospital, Nantong 226001, China

**Keywords:** colorectal cancer, exercise, theory of planned behavior, multicomponent intervention, nudge strategies, quality of life, cancer-related fatigue, patient-reported outcomes, randomized trial

## Abstract

Background: Exercise can reduce symptom burden during colorectal cancer treatment, but the patient-reported effects of multicomponent programs that combine behavioral theory, individualized exercise prescription, and low-burden nudge strategies remain uncertain. Methods: This secondary patient-reported outcome analysis used the completed Shanghai single-center cohort of an open-label, parallel-group randomized trial. Fifty-six adults with stage II–IV colorectal cancer were assigned 1:1 to a 12-week theory-of-planned-behavior-informed multicomponent exercise intervention or an active comparator comprising usual care plus exercise guidance. Participants and interventionists were not blinded; scheduled outcome assessments were completed by nurses unaware of allocation. EORTC QLQ-C30 global health/quality of life and Cancer Fatigue Scale total score were designated as the two primary outcomes of this secondary analysis; they were not prespecified as co-primary outcomes of the parent trial. Baseline-adjusted mixed models for repeated measures estimated week12 effects, with Holm adjustment within this two-outcome family. Results: All 56 randomized participants contributed to longitudinal analyses; week 12 core outcomes were observed for 28 intervention and 27 control participants. The adjusted intervention-control difference was 22.75 points for global health/quality of life (95% CI 14.66–30.84; adjusted *p* < 0.001) and −14.74 points for Cancer Fatigue Scale total score (95% CI −18.48 to −10.99; adjusted *p* < 0.001). QLQ-C30 fatigue was 30.53 points lower, self-efficacy was 3.66 points higher, and adjusted physical activity was approximately doubled (ratio 2.03). No serious exercise-related adverse event was recorded; descriptive muscle-soreness counts were 7/28 versus 16/28 participants. Conclusions: The intervention group showed favorable adjusted 12-week differences in quality of life and fatigue, accompanied by coherent changes in self-efficacy and physical activity. Because delivery was open label and intervention contact exceeded comparator contact, the estimates represent the complete supported program and may include attention and expectation effects. These single-center secondary-analysis estimates support prospective confirmation in a fully enrolled multicenter trial. Trial registration: ChiCTR2400087302, retrospectively registered 24 July 2024. Funding: Shanghai institutional research programs.

## 1. Introduction

Exercise is an evidence-based component of oncology care and is recommended during active cancer treatment to improve function and symptom control [1]. Umbrella and disease-specific syntheses show benefits across cancer populations, including colorectal cancer, while contemporary fatigue guidance identifies exercise as a central nonpharmacologic strategy [2,3,4,5]. Fatigue nevertheless remains persistent and heterogeneous after colorectal cancer diagnosis, emphasizing the need for interventions that patients can sustain during treatment [6].

Treatment-period studies support the feasibility and patient-reported benefits of exercise in colorectal cancer [7,8]. Clinical uptake, however, remains difficult: physical activity is commonly below recommended levels during chemotherapy, and reviews identify symptom burden, competing treatment demands, limited access to tailored advice, and gaps in referral pathways, staffing, and resources as recurring barriers [9,10]. The practical problem is therefore not simply whether exercise works, but how to convert advice into repeated action when fatigue and treatment schedules compete with exercise.

The theory of planned behavior explains action through attitude, subjective norms, perceived behavioral control, and behavioral intention [11]. Research in colorectal cancer and broader cancer populations supports these constructs, together with clinician counseling and self-efficacy, as determinants of physical activity [12,13,14]. Nudge and digital-support strategies can reduce friction through symptom-responsive coaching, reminders, self-monitoring, feedback, and action planning [15,16,17].

This secondary analysis evaluated whether a 12-week, theory-of-planned-behavior-informed multicomponent exercise intervention improved quality of life and cancer-related fatigue and whether these differences were accompanied by coherent behavioral change. EORTC QLQ-C30 global health/quality of life and Cancer Fatigue Scale total score were designated as the two primary outcomes of this secondary analysis, while remaining secondary outcomes in the parent trial; EORTC QLQ-C30 fatigue, exercise self-efficacy, and physical activity provided convergent symptom and behavioral evidence. Exercise- and treatment-related harms were summarized to place benefits in clinical context.

## 2. Materials and Methods

### 2.1. Study Design and Setting

This was a secondary patient-reported outcome analysis of the completed Shanghai single-center cohort within a broader multicenter trial framework, using an open-label, parallel-group design with 1:1 allocation. Participants in the present cohort were recruited at a tertiary hospital in Shanghai, China, from November 2023 to September 2024 and followed through the 12-week end-of-intervention assessment. The present report does not represent completion of the broader planned multicenter enrollment. This secondary report was undertaken after the Shanghai site completed its prespecified 28-per-group accrual and 12-week follow-up, providing a complete site-level randomized cohort for the PRO question rather than an interim subset selected during ongoing site recruitment. The trial was registered in the Chinese Clinical Trial Registry on 24 July 2024, after recruitment had begun and before recruitment was completed, and is therefore retrospectively registered relative to first enrollment (ChiCTR2400087302; https://www.chictr.org.cn/ (accessed on 20 September 2026)). The institutional ethics committee approved the study on 14 July 2023, before participant enrollment (SHSY-IEC-5.0/23K60/P01), and all participants provided written informed consent. The original trial report addressed exercise adherence, self-efficacy, physical function, and safety; the present report focuses on previously collected quality-of-life and fatigue outcomes from the same randomized cohort [18]. Reporting follows CONSORT 2025 and the CONSORT extension for patient-reported outcomes [19,20].

The parent study plan designated exercise adherence as the primary outcome and exercise self-efficacy, physical function, fatigue, and quality of life as secondary outcomes. The two PRO outcomes prioritized in the present report, Holm multiplicity adjustment, and mixed-model estimand were selected at the secondary-analysis stage. No prospectively timestamped secondary-analysis plan documented these choices before the trial data were examined. They are therefore not presented as parent-trial co-primary outcomes or as prospectively prespecified confirmatory analyses, and they were not used to determine the parent-trial sample size. Patient experiences obtained through semistructured interviews contributed to intervention development; patients were not involved in analysis or manuscript preparation.

### 2.2. Participants and Randomization

Adults aged >18 years with pathologically or radiologically confirmed stage II–IV colorectal cancer were eligible while receiving systemic antitumor treatment and after an exercise-safety assessment. Functional eligibility included Eastern Cooperative Oncology Group performance status 0–1 or Karnofsky Performance Status >70, adequate major-organ function, and capacity to participate and provide consent. Key exclusions were another malignancy, participation in another drug trial within four weeks, a physical limitation preventing the planned exercise, or expected survival <3 months.

The treatment population included participants receiving postoperative adjuvant chemotherapy for stage II–III disease and systemic treatment for stage IV or metastatic disease. Baseline records categorized documented treatment intervals as every two or every three weeks; interval data were available for 23 control and all 28 intervention participants. The analysis dataset contained no documented reason for the five missing control group interval entries. It also did not provide longitudinal patient-level data on regimen identity or switching, relative dose intensity, dose reductions, cycle delays or interruptions, or toxicity-directed supportive management. The cohort is therefore characterized as a mixed systemic-treatment population rather than a regimen-specific sample.

Sixty-two candidates were assessed; four declined participation and two were excluded for disease-related factors. Fifty-six participants were randomized 1:1 (28 per group) using a computer-generated simple random sequence prepared by a researcher who was not involved in participant recruitment or intervention delivery. Assignments were placed in sequentially numbered, opaque, sealed envelopes held by a researcher who was not responsible for enrollment; the next envelope was opened only after eligibility had been confirmed and the baseline assessment completed. An age-balancing procedure described in the registry reflected an initial implementation plan but was not used in the conducted trial, which used simple randomization. Available implementation records did not document a contemporaneous reason for not carrying forward the registry-planned age-balancing procedure; we therefore report the discrepancy without assigning an unsupported rationale. Participants and intervention providers were not blinded; scheduled outcome assessments were completed by ward nurses who were unaware of group assignment. The analysis retained randomized assignment for all participants.

### 2.3. Intervention and Comparator

The multicomponent intervention combined an individualized exercise prescription with a 12-week sequence mapped to attitude, subjective norms, perceived behavioral control, and intention-to-action translation. The intervention team comprised the study lead, a gastrointestinal-surgery head nurse, two nursing postgraduate implementers, a physician, an exercise rehabilitation professional, and nurses responsible for follow-up and outcome collection. Components included tailored information, face-to-face and WeChat communication, peer and family norms, commitment, affective framing, default options, feedback, priming, and points-based incentives. Exercise professionals adjusted aerobic, resistance, and flexibility activities to treatment tolerance and adverse reactions, with monthly prescription review and additional adaptation when toxicity changed. Participants received two or three in-hospital teaching sessions, daily reminders, weekly self-monitoring, and weekly telephone or WeChat follow-up.

The active comparator received routine nursing care, treatment-related counseling, an exercise guidance booklet, and a recommendation for moderate-intensity aerobic activity three to five days per week for approximately 30 min per day. Comparator participants also received an exercise diary and monthly follow-up addressing exercise participation and general health education. Because the comparator included an exercise booklet and explicit activity targets, it was treated as an active comparator rather than a no-exercise control.

Implementation was assessed with the 15-item Exercise Prescription Adherence Scale, covering exercise performance, monitoring, and help-seeking [21]. Responses were converted to a percentage of the maximum possible score (0–100). Because this was a self-report implementation measure rather than verified session attendance, it was summarized descriptively at weeks 4, 8, and 12. Concomitant systemic treatment and supportive care were directed by the treating clinical team in both groups independently of randomized assignment; the exercise team did not determine anticancer treatment decisions. Regimens could be modified for clinical response or toxicity. Full intervention and comparator details are provided in Supplementary Table S6.

### 2.4. Outcomes and Assessment Schedule

The present analysis included assessments at baseline and weeks 4, 8, and 12, completed before treatment administration at the corresponding hospital visit. Week 12 was the end-of-intervention endpoint. The parent plan also listed an additional post-intervention assessment; no post-intervention follow-up outcome is analyzed in this secondary report. Global health/quality of life and fatigue were scored from the 30-item simplified-Chinese EORTC QLQ-C30 Version 3.0 using the EORTC linear transformation (0–100); higher global-health scores indicate better status and higher fatigue scores indicate worse symptoms [22,23]. Multidimensional fatigue was measured with the validated 15-item Chinese Cancer Fatigue Scale (CFS-C; total 0–60, higher scores indicate greater fatigue) [24,25]. Exercise self-efficacy used the nine-item Chinese Self-Efficacy for Exercise scale (SEE-C); each item is rated from 0 to 10, and the analysis used the item mean (0–10), equivalent to the 0–90 total divided by nine [26,27]. Physical activity used the seven-item Chinese International Physical Activity Questionnaire-Short Form for the previous seven days, summarized as MET-min/week and modeled as log(1 + MET-min/week) [28].

### 2.5. Statistical Analysis

The parent study’s Shanghai cohort sample size was determined from a 6 min walk-test mean difference of 108.64 m and standard deviation of 110.26 m, with two-sided alpha = 0.05, 90% power, 1:1 allocation, and a 20% allowance for attrition; the resulting target was 28 participants per group. The registry listed a broader multicenter recruitment target of 38 participants per group. The present report is restricted to the completed 28-per-group Shanghai cohort and does not represent the full planned multicenter sample. No separate outcome-specific sample-size calculation was performed for this secondary PRO analysis.

The estimand for the present secondary analysis was the intervention-minus-control difference at week 12 in the randomized population. For each outcome, a baseline-adjusted mixed model for repeated measures included centered baseline-value-by-time and group-by-time terms. Postbaseline observations at weeks 4, 8, and 12 were modeled by generalized least squares with an unstructured within-participant correlation matrix and time-specific variances. Restricted maximum likelihood used all available observations without last-observation-carried-forward replacement, consistent with contemporary guidance for patient-reported outcomes affected by intercurrent events [29]. This secondary-analysis method differs from the parent statistical plan, which proposed repeated-measures analysis of variance or generalized estimating equations and last-observation-carried-forward handling of missing efficacy values; the distinction is reported explicitly rather than describing the mixed model as a prespecified parent-trial analysis.

Holm adjustment was applied to the two primary tests defined for this secondary analysis. This procedure controls familywise error within the selected two-outcome family but does not address the process by which that family was prioritized. QLQ-C30 fatigue was supportive; exercise self-efficacy and IPAQ formed a behavioral family controlled with the Benjamini–Hochberg procedure. Effects are reported with 95% confidence intervals, and standardized effects were oriented so positive values favor intervention. Week 12 HC3 ANCOVA assessed robustness. An exploratory stage-stratified HC3 model for each outcome included the centered baseline outcome, randomized group, stage stratum (stage II–III vs. stage IV), and the group-by-stage interaction; stage-specific effects and nominal interaction *p* values were reported without multiplicity adjustment. To address chance baseline differences without overfitting the small sample, an additional post hoc HC3 model was adjusted for the corresponding baseline outcome, age, body mass index, and stage. Two analyses were added to reproduce the parent statistical plan: outcome-specific LOCF was applied through week 12, followed by Gaussian GEE with treatment, categorical visit, treatment-by-visit interaction, an exchangeable working correlation, and robust sandwich standard errors; classical repeated-measures ANOVA was also fitted to the LOCF-completed four-visit data. These were protocol-concordance sensitivity analyses and did not replace the observed-data MMRM.

A ≥10-point improvement in global health/quality of life or decrease in QLQ-C30 fatigue defined an exploratory responder. This threshold was an interpretive anchor rather than a universal patient-level cutoff. Foundational and subsequent EORTC work shows that meaningful change depends on scale, direction, disease context, and comparison, including advanced colorectal cancer and pooled longitudinal trial settings [30,31,32]. Responder analyses were therefore available-case and explicitly exploratory.

Participants in both groups were instructed to record the same predefined exercise-related symptoms, including muscle soreness, joint discomfort, dizziness, and cardiac discomfort, in exercise diaries throughout the 12-week period. Chemotherapy-related gastrointestinal reactions, myelosuppression, and peripheral neuropathy were abstracted for both groups from routine clinical records at treatment visits and graded with Common Terminology Criteria for Adverse Events Version 5.0 where applicable. The core data sources and event categories were therefore common to both groups, but opportunities for symptom prompting were not identical because intervention participants received weekly contact and comparator participants received monthly follow-up. Adverse events were assessed by the study team; no independent blinded adjudication committee was used. Harms were summarized descriptively by randomized group.

Residual-versus-fitted and normal Q-Q plots were reviewed for both fatigue outcomes, exercise self-efficacy, and log-transformed IPAQ. Observations with large normalized residuals were retained because source records did not support deletion; concordance with HC3 ANCOVA was the primary inferential robustness check. Analyses were conducted in R 4.5.2.

## 3. Results

### 3.1. Participant Disposition and Data Completeness

**Figure 1:**
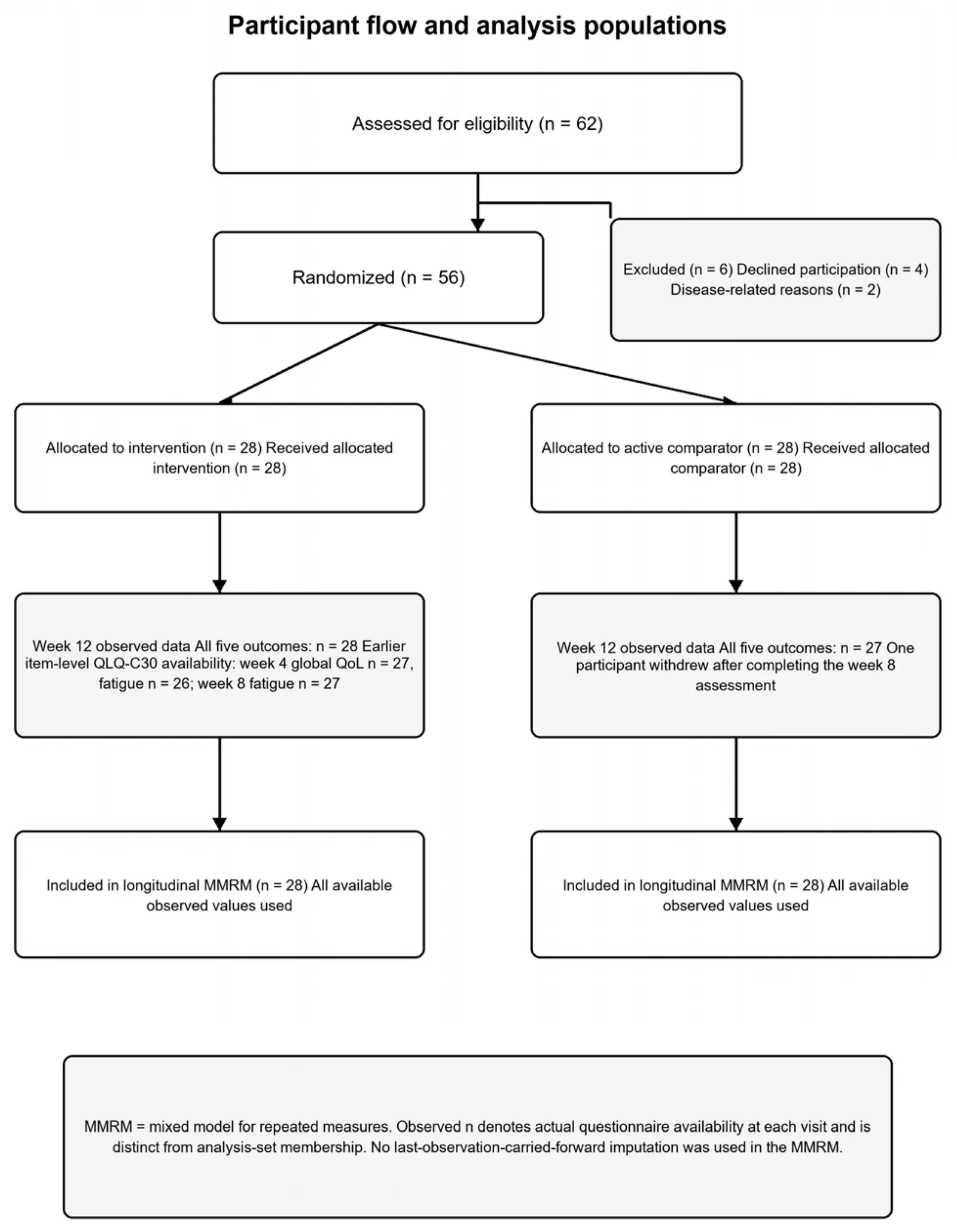
Participant flow and analysis populations. All 56 randomized participants were retained in the longitudinal mixed-model analyses, which used all available observed values without LOCF. Observed *n* values indicate actual questionnaire availability and are distinct from analysis-set membership.

All 56 randomized participants entered the longitudinal analysis. At the end of intervention at week 12, 55 participants (28 intervention and 27 control) had observed global health/quality of life, Cancer Fatigue Scale, exercise self-efficacy, and IPAQ data. QLQ-C30 item-level missingness produced one additional missing global score at week 4 and two missing fatigue scores at week 4 plus one at week 8. Outcome-specific denominators are reported in the text, tables, and participant-flow diagram (Figure 1), as well as Supplementary Table S5.

One control participant withdrew after an observed 8-week assessment; week 8 quality of life, Cancer Fatigue Scale, and exercise self-efficacy were observed values, while week 12 values remained missing. The mixed models used the observed record and did not replace post-withdrawal values.

### 3.2. Baseline Profile

The sample included 11 participants with stage II disease, 35 with stage III disease, and 10 with confirmed stage IV disease. Sex was available for 28 control and 27 intervention participants, and documented treatment interval was available for 23 control and 28 intervention participants (Table 1). Baseline global health/quality of life and exercise self-efficacy were closely aligned, whereas standardized mean differences identified chance differences in age, body mass index, fatigue, residence, education, and occupation. In accordance with randomized-trial reporting, baseline characteristics were described without significance testing; primary models adjusted for the corresponding baseline outcome, and a post hoc sensitivity model additionally adjusted for age, body mass index, and stage.

### 3.3. Patient-Reported Outcomes

At week 12, the adjusted intervention-control difference in global health/quality of life was 22.75 points (95% CI 14.66–30.84; Holm-adjusted *p* < 0.001). The benefit-oriented standardized effect was 0.90 (95% CI 0.58–1.22), and the complete confidence interval exceeded the exploratory 10-point interpretive anchor.

**Figure 2:**
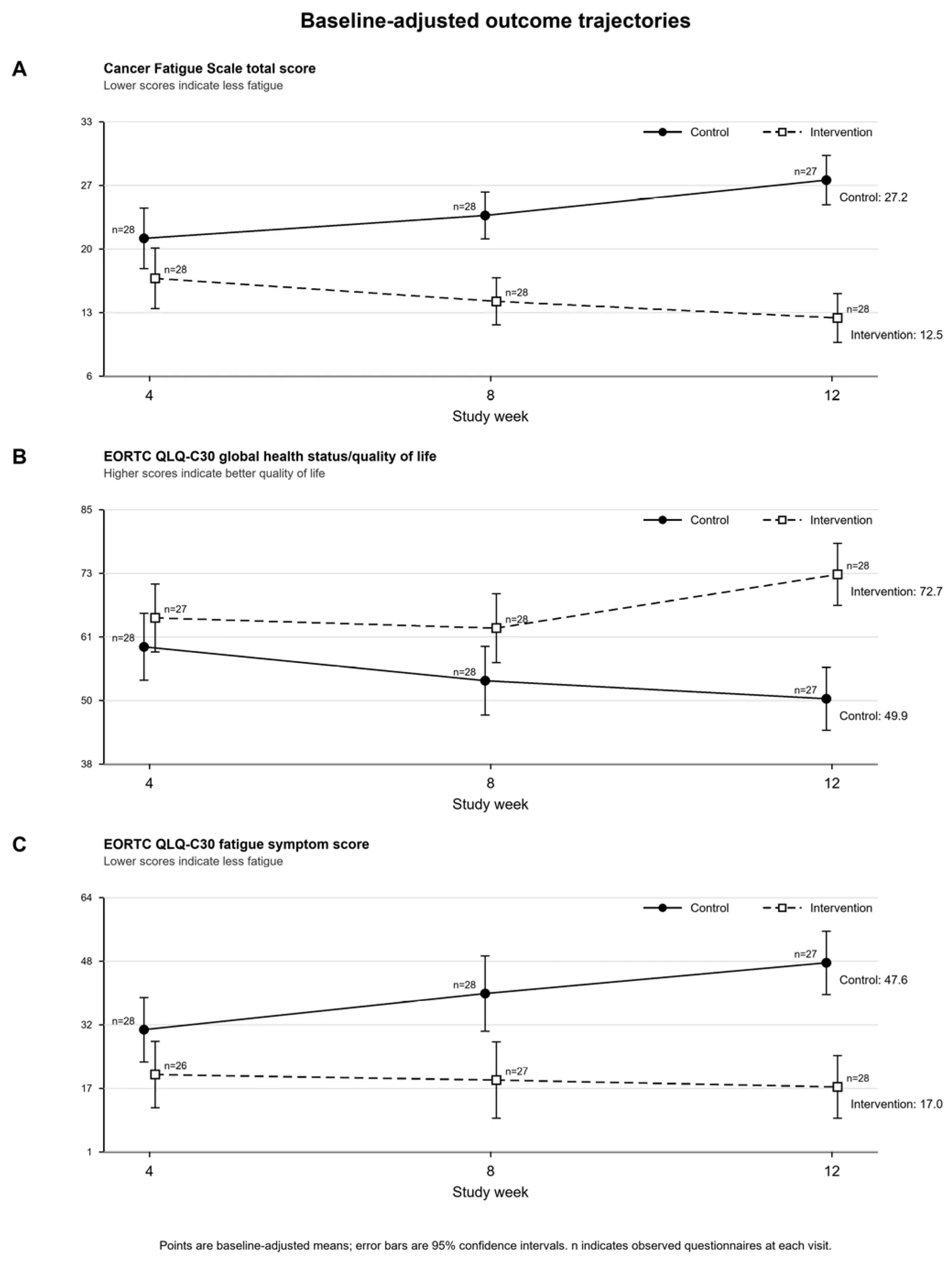
Baseline-adjusted outcome trajectories from the mixed models for repeated measures (MMRM): (**A**) Cancer Fatigue Scale total score; (**B**) EORTC QLQ-C30 global health status/quality of life; and (**C**) EORTC QLQ-C30 fatigue symptom score. Solid lines with filled circles denote the control group; dashed lines with open squares denote the intervention group. Points are baseline-adjusted least-squares means estimated from the MMRM; error bars are 95% confidence intervals; and *n* denotes the number of observed questionnaires at each visit.

**Figure 3:**
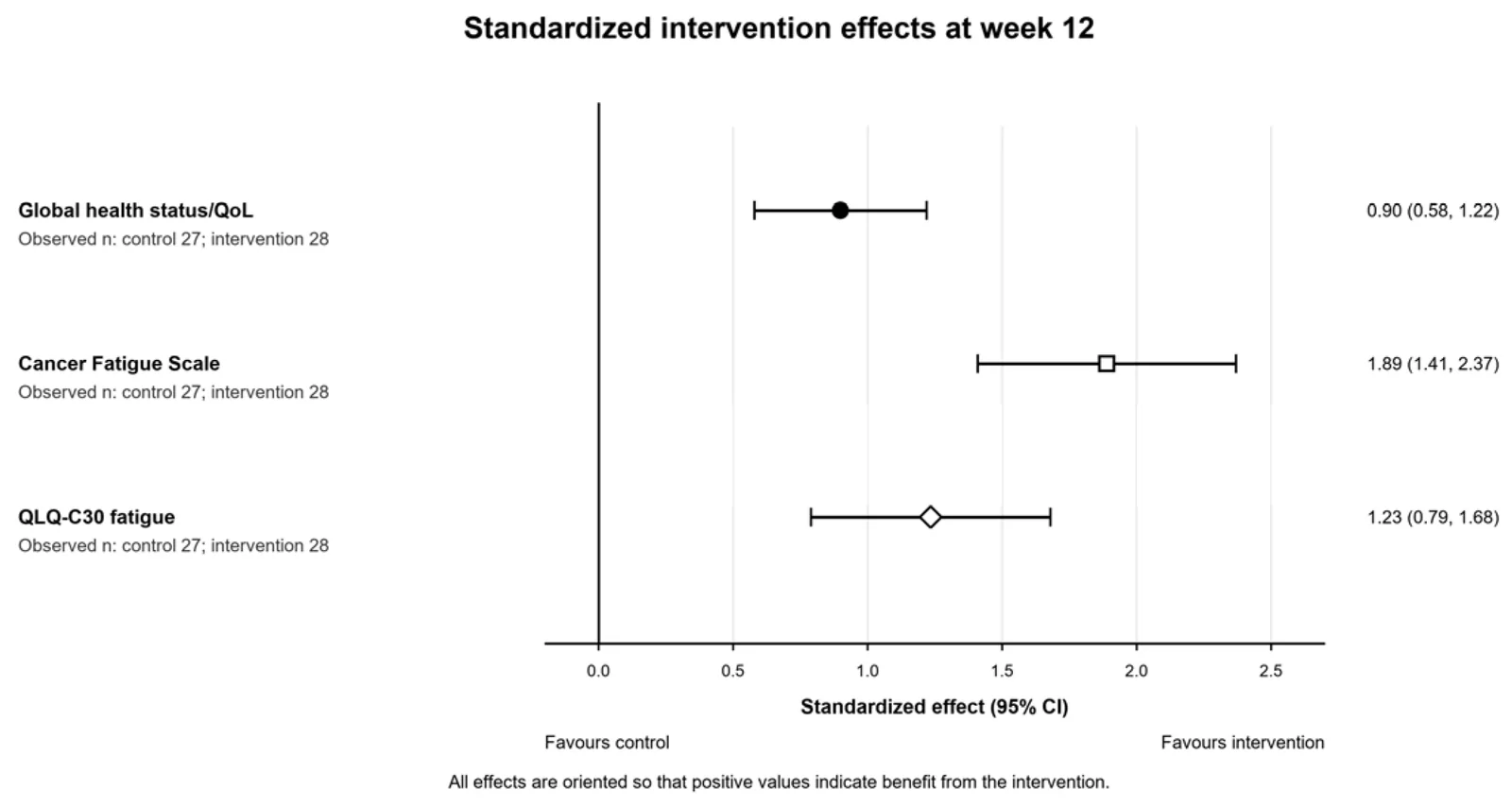
Standardized intervention effects at week 12. Positive values favor the intervention; points show standardized effects and horizontal lines show 95% confidence intervals. Observed sample sizes from week 12: 27 in the control group and 28 in the intervention group.

Cancer Fatigue Scale total score was 14.74 points lower with intervention (95% CI 10.99–18.48; Holm-adjusted *p* < 0.001), equivalent to a benefit-oriented standardized effect of 1.89 (95% CI 1.41–2.37). QLQ-C30 fatigue was 30.53 points lower (95% CI 19.52–41.54; *p* < 0.001), providing convergent evidence across two fatigue instruments (Table 2; Figure 2 and Figure 3).

### 3.4. Clinically Interpretable Response

A ≥10-point improvement in global health/quality of life occurred in 16/28 intervention participants (57.1%) and 4/27 controls (14.8%), giving a risk difference of 42.3 percentage points (95% CI 19.6–65.0), risk ratio 3.86 (95% CI 1.48–10.07), and Fisher exact *p* = 0.002. For QLQ-C30 fatigue, 10/28 (35.7%) versus 4/27 (14.8%) achieved a ≥10-point decrease; the risk difference was 20.9 percentage points (95% CI −1.3 to 43.1). The global-health responder result reinforces the continuous endpoint, whereas the fatigue responder result remains descriptive.

### 3.5. Behavioral Outcomes and Coherence with PRO Change

At week 12, exercise self-efficacy was 3.66 points higher on the 0–10 mean scale (95% CI 3.05–4.27; Benjamini–Hochberg-adjusted *p* < 0.001; standardized effect 1.42). The adjusted geometric mean of IPAQ + 1 was 2.03 times that of control (95% CI 1.56–2.63; adjusted *p* < 0.001).

The self-reported exercise-adherence score rose across the intervention period: the median was 80.8% (IQR 60.0–86.7) at week 4, 85.0% (76.3–92.5) at week 8, and 91.7% (81.3–96.7) at week 12. Corresponding control values were 64.2% (49.6–75.0), 55.0% (48.3–62.1), and 48.3% (39.2–53.3). Week 12 summaries used 28 intervention and 27 observed control assessments; the post-withdrawal value was excluded (Supplementary Table S4).

Exploratory group-adjusted change correlations aligned behavioral activation with symptom benefit. Greater IPAQ improvement correlated with Cancer Fatigue Scale improvement (partial Spearman rho = 0.466) and QLQ-C30 fatigue improvement (rho = 0.467); self-efficacy improvement correlated with both fatigue measures (rho = 0.336 and 0.366). These same-period associations support pathway coherence but are not interpreted as mediation (Supplementary Table S3).

### 3.6. Robustness and Model Diagnostics

Week 12 HC3 ANCOVA closely reproduced the mixed-model estimates: +23.07 points for global health/quality of life, −14.77 for Cancer Fatigue Scale, −31.03 for QLQ-C30 fatigue, +3.65 for self-efficacy, and an IPAQ ratio of 2.05. Additional post hoc adjustment for baseline outcome, age, body mass index, and stage yielded corresponding estimates of +23.56, −14.52, −29.81, and +3.64, as well as an IPAQ ratio of 1.90 (Supplementary Tables S1 and S2). Exploratory stage-stratified estimates favored intervention in both stage II–III and stage IV participants for all five outcomes. Treatment-by-stage interaction *p* values ranged from 0.167 to 0.613; the stage IV estimates were imprecise because this subgroup included 10 participants (Supplementary Table S8). The protocol-concordance GEE/LOCF analysis was similarly aligned: week 12 differences were +22.32 points for global health/quality of life (95% CI 14.22–30.42), −15.54 for Cancer Fatigue Scale (95% CI −19.54 to −11.54), −31.35 for QLQ-C30 fatigue (95% CI −42.48 to −20.21), and +3.64 for self-efficacy (95% CI 2.95–4.33); all *p* < 0.001. Repeated-measures ANOVA on the same LOCF datasets also supported treatment-by-visit interactions (all *p* ≤ 0.002; Supplementary Table S7). Cancer Fatigue Scale residuals closely followed the reference distribution; QLQ-C30 fatigue and self-efficacy showed mild upper-tail departures, and log-transformed IPAQ retained a small number of large residuals (Supplementary Figure S1). Agreement across model classes and missing-data conventions supports the direction of the primary findings.

### 3.7. Safety Context

No serious adverse event was recorded as directly related to exercise. Muscle soreness occurred in 7/28 intervention participants and 16/28 controls; joint pain in 2/28 and 5/28; dizziness in 3/28 and 7/28; and cardiac discomfort in 0/28 and 3/28, respectively. Grade ≥3 myelosuppression, gastrointestinal reactions, and peripheral neuropathy are reported descriptively by randomized group. Because harms were not independently adjudicated, contact frequency differed between groups, and the trial was not powered for between-group safety comparisons—these counts provide clinical context rather than evidence of a protective treatment effect.

## 4. Discussion

### 4.1. Principal Findings

In this secondary PRO analysis, the intervention group showed a more than 22-point adjusted advantage in global health/quality of life and lower fatigue across two instruments at week 12. Both outcomes prioritized for this secondary analysis remained significant after multiplicity adjustment, standardized effects were large, and sensitivity analyses converged. The results form a coherent patient-centered pattern across quality of life, multidimensional fatigue, symptom-specific fatigue, self-efficacy, and physical activity.

A large multinational trial of supervised, structured, and individualized exercise during ongoing oncologic treatment similarly centered baseline-adjusted repeated-measures estimates, multiplicity control, patient-reported fatigue and quality of life, and intervention adherence [33]. The present pattern also extends evidence from colorectal cancer exercise studies delivered during neoadjuvant or active treatment [34,35]. This study applies that patient-centered reporting logic to a shorter behavioral-support program embedded within systemic colorectal cancer treatment.

### 4.2. Why the Combined Strategy Matters

Knowing that exercise is beneficial does not ensure participation during treatment. Attitude-oriented education connected activity with treatment goals; family, peer, and clinical-team cues strengthened subjective norms; individualized prescriptions and symptom-responsive adjustment increased perceived control; and reminders, feedback, commitment, and incentives reduced friction between intention and action. Feasibility work in older or frail patients, metastatic colorectal cancer, and advanced cancer supports adaptive delivery across heterogeneous treatment populations [35,36,37,38].

The rising self-reported adherence percentage, self-efficacy advantage, and twofold physical activity ratio show that implementation and targeted behavioral constructs changed in the expected direction. The adherence measure captures reported enactment rather than objectively verified session attendance, so its role is supportive: it strengthens the intervention-delivery narrative without serving as a causal mediator. A recent randomized study in colon cancer further supports motivation and perceived control as actionable exercise-program endpoints [39,40]. Because behavioral and symptom changes were assessed over the same interval, their associations are interpreted as triangulation rather than formal mediation.

### 4.3. Clinical Interpretation

The global health/quality-of-life estimate is clinically conspicuous: its complete 95% confidence interval lies above 10 points, and the exploratory responder analysis translates the continuous effect into a 42-percentage-point absolute advantage. Scale- and context-specific minimal important differences remain preferable to a universal cutoff [30,31,32], so the 10-point responder is an interpretive lens rather than a confirmatory endpoint. The continuous mixed model remains the primary evidence.

The large, standardized effects should be read together with the raw-scale trajectories and delivery context. From baseline to week 12, mean Cancer Fatigue Scale scores increased by 9.44 points in control and decreased by 3.96 points with intervention; QLQ-C30 fatigue increased by 20.58 points in control and decreased by 4.76 points with intervention, while global health/quality of life decreased by 9.26 points in control and increased by 14.58 points with intervention. The magnitude therefore reflects bidirectional trajectories during active treatment, and standardization against modest baseline dispersion further enlarges the numerical CFS effect. Considered alongside broader exercise-oncology evidence [2,3,33], these conspicuous estimates make delivery context important: the multicomponent intervention provided more counseling, reminders, WeChat contact, feedback, and incentives than the active comparator. In an open-label study with self-reported outcomes, these components may contribute attention, expectation, performance, social-desirability, and Hawthorne effects alongside changes attributable to exercise behavior. The raw differences and confidence intervals are therefore more informative than standardized effects alone, and the estimates should be interpreted as the effect of the complete supported program rather than exercise dose in isolation.

The inclusion of stage II–IV disease reflects registered eligibility and the actual systemic-treatment population, including participants treated for metastatic disease. Exploratory stage-stratified estimates were directionally favorable in both strata, with no interaction *p* value below 0.167, although stage IV estimates were imprecise because that subgroup comprised 10 participants (Supplementary Table S8). This broad clinical frame is consistent with advanced-disease exercise literature [36,37,38], but the analysis does not establish equivalent effects across stages.

### 4.4. Strengths, Boundaries, and Robustness

The secondary analysis combines repeated PRO measurement, high observed-data coverage through week 12, two complementary fatigue instruments, multiplicity control, descriptive adherence reporting, and concordant sensitivity analyses. Mixed models retained all observed values under a missing-at-random framework without carrying post-withdrawal values forward. The parent-plan GEE/LOCF and repeated-measures ANOVA analyses produced closely aligned estimates and treatment-by-visit evidence under a more conservative carried-forward convention. Residual departures are reported transparently, and agreement across HC3, stage-restricted, covariate-adjusted, and protocol-concordance analyses strengthens the directional interpretation without removing the assumptions of any model.

Interpretation is anchored to a 12-week, open-label, single-center cohort of 56 participants drawn from a broader registered multicenter framework. The parent plan treated fatigue and quality of life as secondary outcomes. The outcome hierarchy, Holm adjustment, and mixed-model strategy were selected for this secondary report without a prospectively timestamped secondary-analysis plan documenting those choices before the trial data were examined. Holm adjustment controls familywise error within the selected two-outcome family but cannot eliminate selective-prioritization risk; these estimates are therefore secondary rather than confirmatory trial-level evidence. The trial was retrospectively registered after enrollment began, and the present analysis did not include a post-intervention follow-up; the findings therefore address change during the 12-week intervention and do not establish persistence after support ended. Although LOCF reproduced the parent plan and yielded concordant estimates, it assumes that the last observed value remains applicable at later visits; the likelihood-based observed-data MMRM therefore remains the main analysis. Unequal contact between groups, open-label delivery, self-reported outcomes and behavioral measures, lack of objective exercise-dose verification, chance baseline differences, differential opportunities for exercise-symptom prompting, and the lack of independent adverse-event adjudication bound attribution, magnitude, safety comparison, and generalizability. In a trial of this size, balance cannot be assumed for time-varying treatment intensity, dose modification, cycle delay or interruption, treatment switching, and toxicity management, which were unavailable for covariate adjustment and may have contributed to PRO trajectories. The design therefore cannot separate exercise-specific effects from treatment-exposure differences or from the attention, expectation, and engagement effects of the multicomponent support package.

## 5. Conclusions

In this secondary analysis of a randomized trial, a theory-of-planned-behavior-informed multicomponent exercise intervention delivered during systemic treatment for colorectal cancer was associated with favorable adjusted 12-week differences in global quality of life and cancer-related fatigue. Parallel changes in exercise self-efficacy and physical activity and concordant sensitivity analyses support a coherent supportive-care signal that warrants confirmation in a prospectively registered, fully enrolled multicenter trial.

## Figures and Tables

**Table 1:** Selected baseline characteristics.

Characteristic	Control (*n* = 28)	Intervention (*n* = 28)	Absolute SMD
Age, years, mean (SD)	63.11 (7.03)	59.89 (12.55)	0.316
BMI, kg/m^2^, mean (SD)	23.59 (2.79)	22.78 (2.38)	0.314
Male sex	16/28 (57.1%)	17/27 (63.0%)	0.119
Rectal cancer	11/28 (39.3%)	10/28 (35.7%)	0.074
Stage II/III/IV	6/18/4	5/17/6	Maximum 0.187
Chemotherapy every 2 weeks	9/23 (39.1%)	14/28 (50.0%)	0.220
Chemotherapy every 3 weeks	14/23 (60.9%)	14/28 (50.0%)	0.220
QLQ-C30 global health/QoL, mean (SD)	58.63 (25.10)	58.04 (25.51)	0.024
CFS total, mean (SD)	18.18 (6.38)	15.93 (9.00)	0.288
QLQ-C30 fatigue, mean (SD)	27.38 (25.92)	21.03 (23.49)	0.257
SEE mean (0–10), mean (SD)	4.62 (2.30)	4.60 (2.84)	0.011
IPAQ MET-min/week, median [IQR]	877.5 [448.9, 1680.0]	693.0 [334.9, 1386.0]	0.189 *

SMD, standardized mean difference; BMI, body mass index; QoL, quality of life; CFS, Cancer Fatigue Scale; SEE, exercise self-efficacy; IPAQ, International Physical Activity Questionnaire. * Calculated on log(IPAQ + 1). Sex was missing for one intervention participant; chemotherapy interval was missing for five control participants.

**Table 2:** Week 12 patient-reported outcome effects.

Outcome	Adjusted Intervention-Control Effect (95% CI)	Adjusted *p*	Benefit-Oriented Standardized Effect (95% CI)
QLQ-C30 global health/QoL	+22.75 (14.66 to 30.84)	<0.001 *	0.90 (0.58 to 1.22)
Cancer Fatigue Scale total	−14.74 (−18.48 to −10.99)	<0.001 *	1.89 (1.41 to 2.37)
QLQ-C30 fatigue	−30.53 (−41.54 to −19.52)	<0.001	1.23 (0.79 to 1.68)

Positive standardized effects favor intervention. * Holm-adjusted across the two primary outcomes defined for this secondary analysis.

## Data Availability

The data presented in this study are available on request from the corresponding author. The dataset contains individual-level clinical and patient-reported outcome data from a small single-center oncology cohort. Public deposition was not covered by the participants’ informed consent or institutional ethics approval and could create a risk of participant re-identification. The statistical code and data dictionary supporting this analysis are also available from the corresponding author on reasonable request, subject to institutional approval and applicable ethical and privacy requirements.

## Supplementary Materials

The following supporting information can be downloaded at: https://www.mdpi.com/article/10.3390/healthcare14213771/s1; Table S1, Sensitivity analyses for patient-reported outcomes; Table S2, Sensitivity analyses for supportive behavioral outcomes; Table S3, Exploratory group-adjusted change associations; Table S4, Descriptive self-reported exercise-adherence score by assessment week; Figure S1, Residual diagnostics for repeated-measures models of Cancer Fatigue Scale total score, EORTC QLQ-C30 fatigue, exercise self-efficacy, and log-transformed IPAQ physical activity; Table S5, Observed-data availability and missingness by randomized group and visit; Table S6, TIDieR-structured description of the multicomponent exercise intervention and active comparator; Table S7, Protocol-concordance GEE/LOCF and repeated-measures ANOVA sensitivity analyses; Table S8, Exploratory stage-stratified week-12 effects and treatment-by-stage interaction. The multicomponent intervention and active comparator are described according to the Template for Intervention Description and Replication (TIDieR) framework in Supplementary Table S6 [41].
