# Supplementary material for: Patient-Reported Quality of Life and Fatigue After a Theory-of-Planned-Behavior-Informed Multicomponent Exercise Intervention During Systemic Treatment for Colorectal Cancer: A Secondary Analysis of a Randomized Trial
Source: Healthcare (Basel). 2026 Nov 4;14(21):3771. doi: 10.3390/healthcare14213771 (PMC13762731; doi:10.3390/healthcare14213771)
Supplement: Supplementary file 1 [file healthcare-14-03771-s001.zip › 03_Supplementary_Material_R2.pdf]

Supplementary Material

Patient-Reported Quality of Life and Fatigue after a Theory-of-Planned-Behavior-Informed Multicomponent Exercise Intervention during Systemic Treatment for Colorectal Cancer: A Secondary Analysis of a Randomized Trial

Supplementary Table S1

Sensitivity analyses for patient-reported outcomes

**Outcome**	**Sensitivity analysis**	**Estimate**	**95% CI**	**p**
EORTC QLQ-C30 global health/QoL	Stage II-III MMRM (week-12 contrast)	21.09	11.93 to 30.25	<0.001
EORTC QLQ-C30 global health/QoL	Week-12 HC3 ANCOVA (observed cases)	23.07	14.42 to 31.73	<0.001
Cancer Fatigue Scale total	Stage II-III MMRM (week-12 contrast)	-15.23	-19.30 to -11.16	<0.001
Cancer Fatigue Scale total	Week-12 HC3 ANCOVA (observed cases)	-14.77	-18.65 to -10.88	<0.001
QLQ-C30 fatigue	Stage II-III MMRM (week-12 contrast)	-33.87	-46.20 to -21.54	<0.001
QLQ-C30 fatigue	Week-12 HC3 ANCOVA (observed cases)	-31.03	-42.47 to -19.60	<0.001
EORTC QLQ-C30 global health/QoL	Week-12 HC3 ANCOVA (observed cases; baseline outcome + age + BMI + stage)	23.56	14.75 to 32.36	<0.001
Cancer Fatigue Scale total	Week-12 HC3 ANCOVA (observed cases; baseline outcome + age + BMI + stage)	-14.52	-18.78 to -10.25	<0.001
QLQ-C30 fatigue	Week-12 HC3 ANCOVA (observed cases; baseline outcome + age + BMI + stage)	-29.81	-42.49 to -17.12	<0.001

Note: Effects are intervention minus control; negative values indicate lower fatigue in the intervention group. The stage II-III MMRM is an observed-data subgroup sensitivity analysis, and HC3 ANCOVA uses participants with an observed week-12 outcome. P values are nominal.

Supplementary Table S2

Sensitivity analyses for supportive behavioral outcomes

Outcome	Sensitivity analysis	Transformed effect	95% CI	p
Self-Efficacy for Exercise mean (0-10)	Stage II-III MMRM (week-12 contrast)	Difference 3.76	3.08 to 4.44	<0.001
Self-Efficacy for Exercise mean (0-10)	Week-12 HC3 ANCOVA (observed cases)	Difference 3.65	3.00 to 4.29	<0.001
IPAQ-SF MET-min/week (analyzed as log[1 + MET-min/week])	Stage II-III MMRM (week-12 contrast)	Ratio 1.94	1.45 to 2.59	<0.001
IPAQ-SF MET-min/week (analyzed as log[1 + MET-min/week])	Week-12 HC3 ANCOVA (observed cases)	Ratio 2.05	1.56 to 2.71	<0.001
Self-Efficacy for Exercise mean (0-10)	Week-12 HC3 ANCOVA (observed cases; baseline outcome + age + BMI + stage)	Difference 3.64	2.95 to 4.33	<0.001
IPAQ-SF MET-min/week (analyzed as log[1 + MET-min/week])	Week-12 HC3 ANCOVA (observed cases; baseline outcome + age + BMI + stage)	Ratio 1.90	1.42 to 2.55	<0.001

Note: Self-efficacy effects are intervention-minus-control mean differences. IPAQ effects are ratios of geometric means for IPAQ-SF + 1; ratios greater than 1 favor intervention. HC3 ANCOVA uses participants with an observed week-12 outcome. P values are nominal.

Supplementary Table S3

Exploratory group-adjusted change associations

Behavioral change	PRO improvement	n	Partial Spearman rho	BH-adjusted p
log(IPAQ+1) change	CFS improvement	55	0.466	<0.001
log(IPAQ+1) change	QLQ-C30 fatigue improvement	55	0.467	<0.001
log(IPAQ+1) change	Global QoL improvement	55	0.321	0.020
SEE change	CFS improvement	55	0.336	0.018
SEE change	QLQ-C30 fatigue improvement	55	0.366	0.012
SEE change	Global QoL improvement	55	0.147	0.284

Associations are exploratory, adjusted for randomized group, and based on same-period change; they are not mediation estimates.

Supplementary Table S4

Descriptive self-reported exercise-adherence score by assessment week

Assessment	Control, n	Control median [IQR], %	Intervention, n	Intervention median [IQR], %
Week 4	28	64.2 [49.6, 75.0]	28	80.8 [60.0, 86.7]
Week 8	28	55.0 [48.3, 62.1]	28	85.0 [76.3, 92.5]
Week 12	27	48.3 [39.2, 53.3]	28	91.7 [81.3, 96.7]

The 15-item Exercise Prescription Adherence Scale covers exercise performance, monitoring, and help-seeking; responses were converted to a percentage of the maximum possible score. It is a self-report implementation measure, not objectively verified session attendance. The post-withdrawal week-12 value was excluded.

Supplementary Figure S1

Residual diagnostics for repeated-measures models of Cancer Fatigue Scale total score, EORTC QLQ-C30 fatigue, exercise self-efficacy, and log-transformed IPAQ physical activity. Gray and teal points denote the control and intervention groups, respectively. Dashed lines indicate normalized residuals of +/-2; red curves in residual-versus-fitted panels are LOWESS smooths.

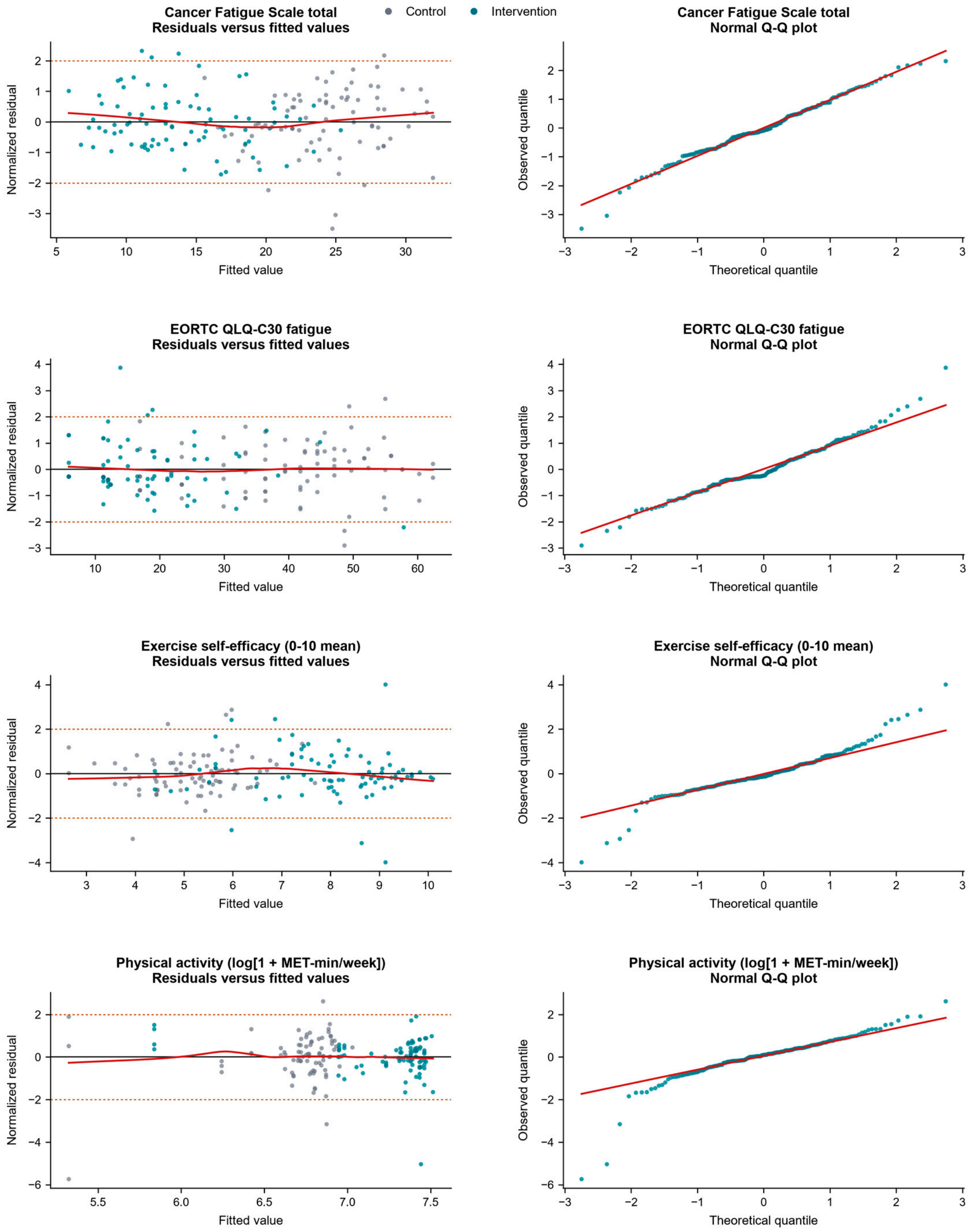

Supplementary Table S5. Observed-data availability and missingness by randomized group and visit

Outcome	Visit	Control observed/N	Control missing	Intervention observed/N	Intervention missing
QLQ-C30 global health/QoL	Baseline	28/28	0	28/28	0
QLQ-C30 global health/QoL	Week 4	28/28	0	27/28	1
QLQ-C30 global health/QoL	Week 8	28/28	0	28/28	0
QLQ-C30 global health/QoL	Week 12	27/28	1	28/28	0
Cancer Fatigue Scale total	Baseline	28/28	0	28/28	0
Cancer Fatigue Scale total	Week 4	28/28	0	28/28	0
Cancer Fatigue Scale total	Week 8	28/28	0	28/28	0
Cancer Fatigue Scale total	Week 12	27/28	1	28/28	0
QLQ-C30 fatigue	Baseline	28/28	0	28/28	0
QLQ-C30 fatigue	Week 4	28/28	0	26/28	2
QLQ-C30 fatigue	Week 8	28/28	0	27/28	1
QLQ-C30 fatigue	Week 12	27/28	1	28/28	0
Exercise self-efficacy mean (0-10)	Baseline	28/28	0	28/28	0
Exercise self-efficacy mean (0-10)	Week 4	28/28	0	28/28	0
Exercise self-efficacy mean (0-10)	Week 8	28/28	0	28/28	0
Exercise self-efficacy mean (0-10)	Week 12	27/28	1	28/28	0
IPAQ-SF (MET-min/week)	Baseline	28/28	0	28/28	0
IPAQ-SF (MET-min/week)	Week 4	28/28	0	28/28	0
IPAQ-SF (MET-min/week)	Week 8	28/28	0	28/28	0
IPAQ-SF (MET-min/week)	Week 12	27/28	1	28/28	0

Note: N is the randomized denominator in each group. One control participant withdrew after an observed week-8 assessment; week-12 values for all five outcomes remained missing and were not replaced in the mixed-model analysis. Additional missingness was limited to EORTC QLQ-C30 item-derived scores in the intervention group: one global health/QoL score at week 4, two fatigue scores at week 4, and one fatigue score at week 8. No other values were missing through week 12. QLQ-C30, European Organisation for Research and Treatment of Cancer Quality of Life Questionnaire Core 30; IPAQ-SF, International Physical Activity Questionnaire-Short Form.

Supplementary Table S6. TIDieR-structured description of the multicomponent exercise intervention and active comparator

TIDieR item	Study-specific description
1. Brief name	Theory-of-planned-behavior-informed multicomponent exercise intervention with nudge-based implementation support.
2. Why	The intervention targeted attitudes, subjective norms, perceived behavioral control, and translation of intention into action during systemic treatment. Nudge components were used to lower practical and motivational barriers to exercise.
3. What: materials	Individualized exercise prescription; exercise guidance booklet; exercise diary; educational articles and case examples; role-model stories; reminder and feedback messages; WeChat step check-ins; and small incentives for achieving agreed exercise goals.
4. What: procedures	Four linked phases were delivered: attitude-oriented education during hospitalization and week 1; commitment, affective, healthcare-professional, and peer support at weeks 1, 3, 5, 7, and 11; individualized prescription, safety planning, feedback, and barrier management across weeks 1-12; and reminders, WeChat step check-ins, feedback, goal setting, and incentives across weeks 4-12.
5. Who provided	A multidisciplinary team included the study lead, a gastrointestinal-surgery nursing lead, a gastrointestinal surgeon, an exercise rehabilitation professional, a gastrointestinal-surgery nurse, and two nursing postgraduate implementers. Clinical and exercise professionals conducted assessment and prescription; nurses and research staff supported delivery and follow-up.
6. How	Delivery combined face-to-face education during hospitalization with telephone and WeChat contact, educational messaging, self-monitoring, feedback, and goal review after discharge.
7. Where	Initial contacts occurred in the inpatient or outpatient setting of a tertiary hospital in Shanghai; remote contacts occurred by telephone or WeChat while participants continued systemic treatment.
8. When and how much	The program lasted 12 weeks. Intervention contacts followed the four-phase schedule described above, including weekly telephone or WeChat review during the implementation period. Exercise dose was individualized to clinical status and treatment tolerance.
9. Tailoring	Exercise prescriptions and goals were individualized. Exercise professionals adjusted aerobic, resistance, and flexibility activities in response to tolerance, adverse reactions, barriers, and changes in clinical status.
10. Modifications	Individual prescription adjustment was built into delivery. Participant-level longitudinal histories of treatment-related exercise modification were not available for this secondary report.
11. Fidelity planned	Exercise diaries, regular telephone or WeChat contacts, step check-ins, goal review, and the 15-item Exercise Prescription Adherence Scale at weeks 4, 8, and 12 were used to support and assess implementation.
12. Fidelity achieved	Self-reported adherence increased in the intervention group from a median of 80.8% at week 4 to 91.7% at week 12; corresponding control values declined from 64.2% to 48.3%. These scores reflect reported enactment rather than device-verified attendance.
Active comparator	Routine nursing care, treatment-related counseling, psychological support as needed, an exercise guidance booklet, advice for moderate-intensity aerobic exercise 3-5 days/week for about 30 min/day and resistance exercise 2-3 times/week for 20-40 min/session, an exercise diary, and periodic follow-up. The comparator therefore contained active exercise guidance.

Note: Structured according to the Template for Intervention Description and Replication (TIDieR): Hoffmann TC, Glasziou PP, Boutron I, et al. Better reporting of interventions: template for intervention description and replication (TIDieR) checklist and guide. BMJ. 2014;348:g1687. doi:10.1136/bmj.g1687. Intervention-delivery descriptions were reconciled with the available study materials; no objective device-based fidelity measure was available.

Supplementary Table S7

Protocol-concordance GEE/LOCF and repeated-measures ANOVA sensitivity analyses

Outcome	LOCF-imputed values, n	GEE/LOCF week-12 effect (95% CI); p	Main MMRM week-12 effect (95% CI)	RM-ANOVA treatment x visit p
QLQ-C30 global health/QoL	2	+22.32 (14.22 to 30.42); <0.001	+22.75 (14.66 to 30.84)	0.001
Cancer Fatigue Scale total	1	-15.54 (-19.54 to -11.54); <0.001	-14.74 (-18.48 to -10.99)	<0.001
QLQ-C30 fatigue	4	-31.35 (-42.48 to -20.21); <0.001	-30.53 (-41.54 to -19.52)	0.002
Exercise self-efficacy (0-10)	1	+3.64 (2.95 to 4.33); <0.001	+3.66 (3.05 to 4.27)	<0.001

Note: Effects are intervention minus control. LOCF was applied outcome by outcome through week 12 solely to reproduce the parent statistical plan. GEE models used a Gaussian identity link, treatment, categorical visit, and treatment-by-visit interaction, an exchangeable working correlation, and robust sandwich standard errors. Classical repeated-measures ANOVA used the same LOCF-completed baseline, week-4, week-8, and week-12 data. The observed-data MMRM remains the main analysis. LOCF-imputed values comprised two global-health/QoL scores, one Cancer Fatigue Scale score, four QLQ-C30 fatigue scores, and one self-efficacy score. GEE, generalized estimating equations; LOCF, last observation carried forward; MMRM, mixed model for repeated measures; RM-ANOVA, repeated-measures analysis of variance.

Supplementary Table S8

Exploratory stage-stratified week-12 effects and treatment-by-stage interaction

**Outcome**	**Stage**	**Observed n C/I**	**Adjusted effect (95% CI)**	**Nominal p**	**Interaction p**
EORTC QLQ-C30 global health/QoL	II-III	23/22	21.63 (12.18 to 31.08)	<0.001	0.550
EORTC QLQ-C30 global health/QoL	IV	4/6	29.46 (4.72 to 54.19)	0.021	0.550
Cancer Fatigue Scale total	II-III	23/22	-15.37 (-19.64 to -11.11)	<0.001	0.167
Cancer Fatigue Scale total	IV	4/6	-9.83 (-16.54 to -3.11)	0.005	0.167
EORTC QLQ-C30 fatigue	II-III	23/22	-34.32 (-47.08 to -21.55)	<0.001	0.203
EORTC QLQ-C30 fatigue	IV	4/6	-12.29 (-43.84 to 19.26)	0.438	0.203
Self-Efficacy for Exercise mean (0-10)	II-III	23/22	3.75 (3.03 to 4.46)	<0.001	0.587
Self-Efficacy for Exercise mean (0-10)	IV	4/6	3.16 (1.14 to 5.18)	0.003	0.587
IPAQ-SF MET-min/week	II-III	23/22	1.96 (1.45 to 2.67)	<0.001	0.613
IPAQ-SF MET-min/week	IV	4/6	2.49 (1.03 to 6.02)	0.043	0.613

Note: Effects are intervention minus control, except IPAQ, which is reported as the ratio of geometric means for IPAQ + 1. Models adjusted for the corresponding baseline outcome and included randomized group, stage stratum (stage II-III vs stage IV), and the group-by-stage interaction, with HC3 standard errors. P values are nominal and were not adjusted for multiplicity. This analysis was exploratory. Stage IV comprised 10 participants (4 control and 6 intervention); interaction tests were underpowered, and a non-significant interaction does not establish equal effects across strata. C/I, control/intervention; IPAQ, International Physical Activity Questionnaire.
