# Supplementary material for: Patient-Reported Quality of Life and Fatigue After a Theory-of-Planned-Behavior-Informed Multicomponent Exercise Intervention During Systemic Treatment for Colorectal Cancer: A Secondary Analysis of a Randomized Trial
Source: Healthcare (Basel). 2026 Nov 4;14(21):3771. doi: 10.3390/healthcare14213771 (PMC13762731; doi:10.3390/healthcare14213771)
Supplement: Supplementary file 1 [file healthcare-14-03771-s001.zip › 04_CONSORT_2025_Checklist_R2.pdf]

CONSORT 2025 checklist of information to include when reporting a randomised trial*

Manuscript ID: healthcare-4621147

Manuscript title: Patient-Reported Quality of Life and Fatigue after a Theory-of-Planned-Behavior-Informed Multicomponent Exercise Intervention during Systemic Treatment for Colorectal Cancer: A Secondary Analysis of a Randomized Trial

Section / Topic	No	CONSORT 2025 checklist item description	Reported on page no.
**Title and abstract**			
Title and structured abstract	1a	Identification as a randomised trial	p. 1, lines 2-6 (title)
	1b	Structured summary of the trial design, methods, results, and conclusions	p. 1, lines 18-39 (structured abstract)
**Open science**			
Trial registration	2	Name of trial registry, identifying number (with URL) and date of registration	p. 1, lines 15-16; p. 2, lines 75-78
Protocol and statistical analysis plan	3	Where the trial protocol and statistical analysis plan can be accessed	Available Chinese-language parent-study statistical analysis plan supplied separately; secondary-analysis design and

Section / Topic	No	CONSORT 2025 checklist item description	Reported on page no.
			methods: pp. 3-5, lines 84-200
Data sharing	4	Where and how the individual de-identified participant data (including data dictionary), statistical code and any other materials can be accessed	p. 13, lines 404-410 (Data Availability Statement)
Funding and conflicts of interest	5a	Sources of funding and other support (e.g., supply of drugs), and role of funders in the design, conduct, analysis and reporting of the trial	p. 12, lines 391-398 (Funding); funding also summarized on p. 1, line 39
	5b	Financial and other conflicts of interest of the manuscript authors	p. 13, lines 413-414 (Conflicts of Interest)
**Introduction**			
Background and rationale	6	Scientific background and rationale	p. 2, lines 44-59
Objectives	7	Specific objectives related to benefits and harms	p. 2, lines 60-66
**Methods**			
Patient and public involvement	8	Details of patient or public involvement in the design, conduct and reporting of the trial	p. 3, lines 89-91 (patient interviews informed

Section / Topic	No	CONSORT 2025 checklist item description	Reported on page no.
			intervention development)
Trial design	9	Description of trial design including type of trial (e.g., parallel group, crossover), allocation ratio, and framework (e.g., superiority, equivalence, non-inferiority, exploratory)	p. 2, lines 69-75 (open-label, parallel-group, 1:1 randomized design and complete Shanghai cohort)
Changes to trial protocol	10	Important changes to the trial after it commenced including any outcomes or analyses that were not prespecified, with reason	p. 3, lines 84-88 and 110-113; pp. 4-5, lines 164-182 (secondary-analysis and implementation departures reported)
Trial setting	11	Settings (e.g., community, hospital) and locations (e.g., countries, sites) where the trial was conducted	p. 2, lines 69-72 (tertiary hospital in Shanghai, China)

Section / Topic	No	CONSORT 2025 checklist item description	Reported on page no.
Eligibility criteria	12a	Eligibility criteria for participants	p. 3, lines 93-98
	12b	If applicable, eligibility criteria for sites and for individuals delivering the interventions (e.g., surgeons, physiotherapists)	pp. 3-4, lines 118-139; Supplementary Table S6, p. 6
Intervention and comparator	13	Intervention and comparator with sufficient details to allow replication. If relevant, where additional materials describing the intervention and comparator (e.g., intervention manual) can be accessed	pp. 3-4, lines 118-139; Supplementary Table S6, p. 6
Outcomes	14	Pre-specified primary and secondary outcomes, including the specific measurement variable (e.g., systolic blood pressure), analysis metric (e.g., change from baseline, final value, time to event), method of aggregation (e.g., median, proportion), and time point for each outcome	p. 4, lines 141-172
Harms	15	How harms were defined and assessed (e.g., systematically, non-systematically)	p. 5, lines 188-196
Sample size	16a	How sample size was determined, including all assumptions supporting the sample size calculation	p. 4, lines 152-158
	16b	Explanation of any interim analyses and stopping guidelines	Not applicable: no interim analysis or statistical stopping guideline was used
Randomisation:			
Sequence generation	17a	Who generated the random allocation sequence and the method used	p. 3, lines 106-116 (computer-

Section / Topic	No	CONSORT 2025 checklist item description	Reported on page no.
			generated sequence; independent generator)
	17b	Type of randomisation and details of any restriction (e.g., stratification, blocking and block size)	p. 3, lines 106-116 (simple randomization without blocking or stratification; registry discrepancy reported)
Allocation concealment mechanism	18	Mechanism used to implement the random allocation sequence (e.g., central computer/telephone; sequentially numbered, opaque, sealed containers), describing any steps to conceal the sequence until interventions were assigned	p. 3, lines 106-116 (sequentially numbered, opaque, sealed envelopes opened after

Section / Topic	No	CONSORT 2025 checklist item description	Reported on page no.
			baseline assessment)
Implementation	19	Whether the personnel who enrolled and those who assigned participants to the interventions had access to the random allocation sequence	p. 3, lines 106-116 (roles in sequence generation, enrollment, concealment, and assignment)
Blinding	20a	Who was blinded after assignment to interventions (e.g., participants, care providers, outcome assessors, data analysts)	p. 1, lines 23-24; p. 3, lines 114-116
	20b	If blinded, how blinding was achieved and description of the similarity of interventions	Not applicable to participants and intervention providers because delivery was open label; scheduled outcome

Section / Topic	No	CONSORT 2025 checklist item description	Reported on page no.
			assessments were completed by nurses unaware of allocation (p. 3, lines 114-116)
Statistical methods	21a	Statistical methods used to compare groups for primary and secondary outcomes, including harms	pp. 4-5, lines 159-200
	21b	Definition of who is included in each analysis (e.g., all randomised participants), and in which group	p. 3, line 116; pp. 4-5, lines 159-182 and 202-210
	21c	How missing data were handled in the analysis	pp. 4-5, lines 163-168, 178-182, and 203-210; Supplementary Tables S5 and S7, pp. 5 and 7

Section / Topic	No	CONSORT 2025 checklist item description	Reported on page no.
	21d	Methods for any additional analyses (e.g., subgroup and sensitivity analyses), distinguishing prespecified from post-hoc	p. 5, lines 173-187; pp. 9-10, lines 252-286; Supplementary Tables S1, S2, S7, and S8, pp. 1, 2, 7, and 8
**Results**			
Participant flow, including flow diagram	22a	For each group, the numbers of participants who were randomly assigned, received intended intervention, and were analysed for the primary outcome	pp. 5-6, lines 202-215 and Figure 1
	22b	For each group, losses and exclusions after randomisation, together with reasons	pp. 5-6, lines 207-215 and Figure 1
Recruitment	23a	Dates defining the periods of recruitment and follow-up for outcomes of benefits and harms	p. 2, lines 71-78; p. 5, lines 202-210
	23b	If relevant, why the trial ended or was stopped	Not applicable: the completed 12-week Shanghai cohort

Section / Topic	No	CONSORT 2025 checklist item description	Reported on page no.
			was not stopped early
Intervention and comparator delivery	24a	Intervention and comparator as they were actually administered (e.g., where appropriate, who delivered the intervention/comparator, how participants adhered, whether they were delivered as intended [fidelity])	pp. 3-4, lines 118-139; p. 9, lines 262-265; Supplementary Tables S4 and S6, pp. 3 and 6
	24b	Concomitant care received during the trial for each group	pp. 3-4, lines 99-105 and 136-139
Baseline data	25	A table showing baseline demographic and clinical characteristics for each group	pp. 6-7, lines 216-228 (Table 1)
Numbers analysed, outcomes and estimation	26	For each primary and secondary outcome, by group:<br>● the number of participants included in the analysis<br>● the number of participants with available data at the outcome time point<br>● result for each group, and the estimated effect size and its precision (such as 95% confidence interval)<br>● for binary outcomes, presentation of both absolute and relative effect size	pp. 5-10, lines 202-286; Table 2 and Figures 1-3; Supplementary Tables S1-S5, pp. 1-5

Section / Topic	No	CONSORT 2025 checklist item description	Reported on page no.
Harms	27	All harms or unintended events in each group	p. 5, lines 188-196; p. 10, lines 287-293
Ancillary analyses	28	Any other analyses performed, including subgroup and sensitivity analyses, distinguishing pre-specified from post-hoc	p. 5, lines 173-187; pp. 9-10, lines 252-286; Supplementary Tables S1-S3, S7, and S8 and Supplementary Figure S1
**Discussion**			
Interpretation	29	Interpretation consistent with results, balancing benefits and harms, and considering other relevant evidence	pp. 10-11, lines 294-343
Limitations	30	Trial limitations, addressing sources of potential bias, imprecision, generalisability, and, if relevant, multiplicity of analyses	pp. 11-12, lines 344-369

*We strongly recommend reading this statement in conjunction with the CONSORT 2025 Explanation and Elaboration and/or the CONSORT 2025 Expanded Checklist for important clarifications on all the items. We also recommend reading relevant CONSORT extensions. See www.consort-spirit.org.

Citation: Hopewell S, Chan AW, Collins GS, Hróbjartsson A, Moher D, Schulz KF, et al. CONSORT 2025 Statement: updated guideline for reporting randomised trials. BMJ. 2025; 388:e081123. https://dx.doi.org/10.1136/bmj-2024-081123.

© 2025 Hopewell et al. This is an Open Access article distributed under the terms of the Creative Commons Attribution License (https://creativecommons.org/licenses/by/4.0/), which permits unrestricted use, distribution, and reproduction in any medium, provided the original work is properly cited.
